# MATN3 Gene Polymorphism Is Associated with Osteoarthritis in Chinese Han Population: A Community-Based Case-Control Study

**DOI:** 10.1100/2012/656084

**Published:** 2012-08-22

**Authors:** Jiaao Gu, Jiesheng Rong, Fulin Guan, Liying Jiang, Shuqing Tao, Guofa Guan, Tianzun Tao

**Affiliations:** ^1^Department of the 2nd Affiliated Hospital of Harbin Medical University, Harbin Medical University, Heilongjiang, Harbin 150086, China; ^2^Department of Epidemiology, Public Health College, Harbin Medical University, Heilongjiang, Harbin 150086, China; ^3^The 1st Affiliated Hospital of Harbin Medical University, Harbin Medical University, Heilongjiang, Harbin 150086, China

## Abstract

*Background*. The matrilin, especially matrilin-3 (MATN3), are reported to play important roles in the pathophysiology of osteoarthritis (OA). To explore the relationship between MATN3 SNP6 (rs8176070) and primary OA, we conducted a community-based case-control study. *Methods*. A total of 732 community residents aged 40–84 years participated in the community-based study in Northeast China. After taking physical and radiographic examinations, 420 of the residents were diagnosed OA (216 women and 204 men). The other 312 individuals without any symptoms of osteoarthritis or signs in the radiographs (156 women and 156 men) were considered as healthy controls. After obtaining the DNA of case and control groups, genotypes of the MATN3 SNP6 were determined by polymerase chain reaction followed by restriction enzyme digestion. The numbers of patients with different OA subtypes were also calculated. *Results*. The distribution of genotypes and alleles of the MATN3 SNP6 between OA patients and controls was different significantly. The BB carrier tends to be associated with the increased osteoarthritis (*P* = 0.025, OR = 1.724, 95% CI = 1.071–2.77), especially the knee osteoarthritis (*P* = 0.021, OR = 2.402, 95% CI = 1.141–5.060) and lumber osteoarthritis (*P* = 0.020, OR = 1.880, 95% CI = 1.103–3.204). Bb carrier increased hand osteoarthritis risk (*P* = 0.002, OR = 5.380, 95% CI = 1.828–15.835). The B allele might have an effect on the increased knee osteoarthritis (*P* = 0.000, OR = 3.143, 95% CI = 2.283–4.328). *Conclusion*. These findings suggest that the MATN3 gene polymorphism might be associated with osteoarthritis in the Chinese Han population.

## 1. Introduction

Osteoarthritis (OA) is a common degenerative joint disease among the elderly; it is also one of the major public health problems [1]. Clinically, the predominant symptoms of osteoarthritis are joint pain, limitation of movement, tenderness, crepitus, occasional effusion, and variable degrees of local inflammation, but without systemic effects [2, 3]. Many predisposing factors may contribute to osteoarthritis progression [4, 5], such as advanced age, genetic, obesity, bone density, hormone level, mechanical factors, past history of trauma, and genetic susceptibilities. Recent studies have also revealed a role of the inflammatory process in the pathogenesis of OA [6–8]. Furthermore, it has been reported that the occurrence and development of OA may be influenced by multiple genes. A number of candidate genes have been suggested to mediate the susceptibility to OA, including collagen genes (COL1A1, COL2A1, COL9A1, and COL11A2), the genes encoding cartilage matrix protein 1 (CMP1), vitamin D receptor (VDR), insulin-like growth factor-1 (IGF1), transforming growth factor-*β*1 (TGF*β*1), aggrecan-1 (AGC1), tissue inhibitor of metalloproteinase 3 (TIMP3), interleukin-1 receptor (IL1R), the estrogen receptor, and cyclooxygenase-2 (COX-2) genes [9–14].

The matrilin (MATNs) are a family of oligomeric extracellular matrix (ECM) proteins consisting at least of four related proteins, termed matrilin-1 through -4 containing. The MATNs contain common structural motifs including von Willebrand factors A (vWFA) domains, epidermal growth factor (EGF)-like domains, and coiled-coil domains [15]. Matrilin-3 (MATN3) is the least complex member of the matrilin family, consisting of only one vWFA domain, four EGF-like domains, and a C-terminal coiled-coil domain. In humans, nine missense mutations in the matrilin-3 gene (MATN3) that affect the vWFA domain (typically the *β*-sheets) have been found in patients with multiple epiphyseal dysplasia (MED), characterized by delayed and irregular ossification of the epiphyses and early-onset osteoarthritis [16–19]. Mutations in MATN3 have also been reported in other osteochondrodysplasias, including bilateral hereditary microepiphyseal dysplasia, a MED-like disorder characterized by small epiphyses in the hip and knee joints, spondyloepimetaphyseal dysplasia, which includes a number of conditions associated with vertebral, epiphyseal, and metaphyseal anomalies [20], and idiopathic hand osteoarthritis [21]. It has been found that enhanced matrilin-3 gene and protein expression was correlated with the extent of tissue damage in osteoarthritis patients [22]. These findings suggest that tight regulation of matrilin-3 expression is essential for maintenance of the cartilage ECM microenvironment. 

Recently, few studies aimed to investigate the relationship between a functional single-nucleotide polymorphism (SNP) in the MATN3 (rs8176070) and OA, while the results were inconsistent. Two studies found the mutation of SNP6 was associated with susceptibility to hand osteoarthritis in the Caucasian population sample [21, 23]. One found the variation of MATN3 was not associated with OA in the European population [24]. Our objective was to assess the relationship between MATN3 rs8176070 (SNP6) and primary OA (including cervical, lumber, and knee and hand osteoarthritis) in Chinese Han population.

## 2. Materials and Methods

### 2.1. Study Subjects

The study was performed in Heilongjiang province, a northeast region, from 2005 to September 2009. The study population was drawn from the population aged 40 years and over living in communities using stage-stratified sampling methods. First, Xiangfang district representing the middle economic level for urban areas of Heilongjiang province was selected from 7 districts of Harbin, then 4 of 19 communities were randomly selected, and all the residents of sampled communities were investigated. Finally, a total of 800 people were selected and invited to participate in the study; 732 people completed the survey and donated their blood samples. All these people were examined at the 2nd Affiliated Hospital of Harbin Medical University, Heilongjiang, Harbin, China. All these people took radiographs of cervical, lumber, knees, and hands. Radiographs, including anterior-posterior hand and lateral and anterior-posterior cervical, lumber, and knee radiographs, were read by two radiologists blindly and independently who were unaware of the clinical findings until a consensus was reached. People with no sign or symptom of osteoarthritis, other arthritis or joint diseases at any site according to their medical history (such as pain, swelling, tenderness, or restriction of movement), and with no sign in the 7 radiographs, were considered as control group. At the same time, people were recruited into OA group with symptoms or signs of OA and radiographic signs of OA according to the Kellgren-Lawrence grading. Patients with inflammatory arthritis (rheumatoid or autoimmune disease), posttraumatic, or postseptic arthritis were excluded. Demographic and other information including stage, type, onset age, and family history were obtained by using questionnaires. Both patients and controls in this study were Han ethnicity and were all free from systemic, chronic, autoimmune, allergic, and anti-inflammatory diseases. Finally, a total of 420 patients with OA (216 women and 204 men) and 312 healthy controls (156 women and 156 men) were enrolled in the study.

This study was conducted in compliance with the guidelines of the Declaration of Helsinki. All participates were given written informed consent before their participation in this study.

The Kellgren-Lawrence grade represents disease severity reflecting on radiographs. Radiographic findings of OA were classified into mild (Kellgren-Lawrence grade 1 or 2) and severe (Kellgren-Lawrence grade 3 or 4). The functional or symptomatic statuses of OA patients were classified as functionally or symptomatically good (Lequesne's functional index = 10) and poor (Lequesne's functional index > 10) (showed in Table 1) [25]. 

## 3. Analysis of MATN3 Polymorphism

### 3.1. Deoxyribonucleic Acid (DNA) Isolation

Blood samples from patients and controls were collected by Vacutainer tubes (Becton, Dickinson and Company, Franklin Lakes, NJ, USA) and were transferred to EDTA tubes. DNA was extracted from the peripheral blood leucocytes by standard phenol/chloroform extraction techniques and precipitation with ethanol.

### 3.2. MATN3 Genotyping

Genotyping of MATN3 SNP6 was assayed with the polymerase chain reaction restriction fragment length polymorphism (PCR-RFLP). The polymerase chain reaction (PCR) was conducted in a reaction volume of 25 *μ*L with 10 ng genomic DNA, 10 × PCR buffer, 100 *μ*M dNTPs, 5 pmol of each primer, and 0.5 unit of Taq polymerase (Neurotics Inc., Seoul, Republic of Korea). The PCR primers used 5′-GGACAGGATCCCACAAAAAG-3′ as a forward primer and 5′-GAAAGAGGGGCTACAACAGG-3′ as a reverse primer. Amplification was carried out as follows: 35 cycles consisting of 1 min of denaturation at 95°C, 1 min of annealing at 61°C, 1 min of extension at 72°C with an initial denaturation step of 10 min at 95°C, and a final extension of 10 min at 72°C in a thermocycler (Techne, TC-312, UK). The resultant PCR products showed single fragment at 501 bp. 10 *μ*L of 501-bp product were then digested with 10 units of BSEYI restriction enzyme (NEB-R0635S) at 37°C for 10 h. Digestion products were visualized on a 3% agarose gel containing ethidium bromide. The RFLPs were coded as Bb, where the uppercase letter signifies the absence of the restriction site and the lowercase letter signifies the presence of the site. Wild-type genotype (CC) which coded as bb produced double band at 149 and 352 bp, heterozygotes (CN) which coded as Bb produced three bands at 501, 149 and 352 bp, and homozygote polymorphic genotype (NN) which coded as BB produced only one band at 501 bp.

### 3.3. Statistical Analysis

In this case-control study, we used Pearson's chi-square test to determine the significance of differences in allelic and genotypic frequencies between osteoarthritis patients and control groups. *P* < 0.05 was considered statistically significant. Odds ratios (ORs) with 95% confidence intervals (CIs) were also calculated. Hardy-Weinberg equilibrium test was performed by using a *χ* ^2^ test. All statistical analyses were performed with SPSS version 15.0 software (SPSS Inc., Chicago, IL, USA).

## 4. Results

Frequencies of genotypes and alleles of MATN3 SNP6 in patients and controls were calculated. We categorized our osteoarthritis cases into lumbar OA, knee OA, cervical OA, and hand OA. Among all patients, 108 patients with knee OA were in the age ranged from 40 to 79 with a mean age of 60.22 ± 6.80 years, 286 patients with lumbar OA ranged from 42 to 84 with a mean age of 63.45 ± 8.13 years, 228 patients with cervical OA ranged from 41 to 81 with a mean age of 62.23 ± 9.42 years, and 28 patients with hand OA ranged from 53 to 79 with a mean age of 63.22 ± 7.80 years.

We analyzed the frequency of the MATN3 SNP6 in 420 patients and 312 controls. The genotype distribution exhibited a significant difference between OA patients and control groups. The genotype of BB increased the risk of OA (odds ratio [OR] = 1.724, 95% confidence interval [CI] = 1.071–2.770; *P* = 0.025), especially the knee OA (OR = 2.402, 95% CI = 1.141–5.060; *P* = 0.021) and the lumber OA (OR = 1.880, 95% CI = 1.103–3.204; *P* = 0.020). The genotype of Bb was a risk factor for hand OA (OR = 5.380, 95% CI = 1.828–15.835; *P* = 0.002), but not for cervical OA. 

Regarding SNP6, allele frequencies of b and B were 67.9% (424/624) and 32.1% (200/624) in the control group. Allele frequency was significantly different among knee OA patients compared with control individuals (OR = 3.143, 95% CI = 2.283–4.328; *P* = 0.000), while there was no significant difference between controls and the OA, as well as lumber, cervical, and hand OA (Tables 2, 3, 4, 5, and 6).

## 5. Discussion 

Osteoarthritis is the most common form of arthritis. It is the foremost cause of disability in the elderly population, affecting approximately 10% of population over the age of 60 years [26]. In OA, the earliest histological changes involve edema in the articular cartilage, suggesting an alteration in the balance between the proteoglycan and collagen networks. Articular cartilage is mostly made of extracellular-specialized collagens and proteoglycans. The physical properties of articular cartilage support frictionless movements and the load-bearing capacity of the joints [27]. The role of MATN3 in cartilage homeostasis was demonstrated. Some researchers found that functional knockout of MATN3 in mice could induce premature hypertrophy of articular chondrocytes which could contribute to the development of osteoarthritis in adult mice [28].

To our knowledge, the previous studies only investigated the relationship between MATN3 polymorphism and hand OA or knee OA [21, 23, 24]. Two articles claimed that the SNP6 variation is associated with hand OA, but not knee [21, 23], while another article claimed that the mutation of SNP6 is not associated with OA [24]. Our study is the first to analyze the distribution of polymorphisms of MATN3 gene in a group of Chinese osteoarthritis patients and unrelated healthy controls. In this study, we replicated that the mutation of SNP6 is associated with hand OA, as well as lumber OA and knee OA. Contrary to the anterior researches, we found the distribution of SNP6 genotypes had some relationship with knee OA, which might contribute to the ethnicity variation. 

We investigate the MATN3 SNP6 in the patients with OA, and we divided the patients suffering from different sites into different subgroups: knee OA, lumber OA, cervical OA, and hand OA as well. So our results better evaluate the relationship between the MATN3 polymorphism and osteoarthritis, especially the subtype of OA.

Present study has some limitations that should be considered. Firstly, our sample size was relatively small, which may limit statistical power to detect any existing association, especially the number of patients with hand OA. The limited sample size might lead to the distribution of genotype and allele might departure from the real condition. Secondly, the control of MATN3 expression and activity in vivo is complex and not well understood, and could be subjected to modulation by polymorphisms in other genes. Thirdly, lack of original data for each patient with his or her Kellgren-Lawrence grade limited our further analysis of the relationship between the MATN3 gene polymorphism and the severity of osteoarthritis. Moreover, the patients were all from the northeast region of China, so our results might not represent the whole condition of China.

In conclusion, our results suggest that the investigated polymorphism in the MATN3 gene might play a role in osteoarthritis in the Han population. Further study with a larger population size remains to be conducted for a definitive affirmation of correlates of MATN3 gene polymorphism in osteoarthritis patients.

## Figures and Tables

**Table 1:** Clinical characteristics of the osteoarthritis patients and control group.

Characteristic	OA	Normal
Age (years)	57.4 ± 9.5	58.7 ± 9.5
Number women/men (*n*)	216/204	156/156
Body mass index (kg/m^2^)	20.1 ± 8.2	19.9 ± 8.3
Kellgren-Lawrence grade (*n*): 1/2/3/4	32/186/154/48	
Lequesne's index	12.2 ± 2.5	

A total of 420 patients with primary osteoarthritis and 312 control group were included. Values are expressed as mean ± standard deviation or as numbers.

**Table 2:** Genotype and allele frequencies and odds ratios of MATN3 SNP6 (rs8176070) in control, osteoarthritis cases.

	Normals	OA	*P* ^ a^	OR (95% CI)
Genotype	*n* = 312 (%)	*n* = 420 (%)		
bb	149 (47.8)	187 (44.5)		
Bb	126 (40.4)	165 (39.3)	0.414	1.149 (0.823–1.603)
BB	37 (11.9)	68 (16.2)	0.025	1.724 (1.071–2.77)
Allele	*n* = 624 (%)	*n* = 840 (%)		
b	424 (67.9)	539 (64.2)		
B	200 (32.1)	301 (35.8)	0.131	0.845 (0.678–1.052)

*n*: number of samples.

^a^ *P* < 0.05, significantly different, compared with the control group.

**Table 3:** Genotype and allele frequencies and odds ratios of MATN3 SNP6 (rs8176070) in control, knee OA cases.

	Normal	All knee OA	*P* ^ a^	OR (95% CI)
Genotype	*n* = 312 (%)	*n* = 108 (%)		
bb	37 (11.9)	19 (17.6)		
Bb	126 (40.4)	49 (45.4)	0.061	1.662 (0.976–2.830)
BB	149 (47.8)	40 (37.3)	0.021	2.402 (1.141–5.060)
Allele	*n* = 624 (%)	*n* = 216 (%)		
b	424 (67.9)	129 (59.7)		
B	200 (32.1)	87 (40.3)	0.000	3.143 (2.283–4.328)

*n*: number of samples.

^a^ *P* < 0.05, significantly different, compared with the control group.

**Table 4:** Genotype and allele frequencies and odds ratios of MATN3 SNP6 (rs8176070) in control, lumbar OA cases.

	Normal	All lumber OA	*P* ^ a^	OR (95% CI)
Genotype	*n* = 312 (%)	*n* = 286 (%)		
bb	149 (47.8)	122 (42.7)		
Bb	126 (40.4)	117 (40.9)	0.177	1.297 (0.889–1.892)
BB	37 (11.9)	47 (16.4)	0.020	1.880 (1.103–3.204)
Allele	*n* = 624 (%)	*n* = 572 (%)		
b	424 (67.9)	361 (63.1)		
B	200 (32.1)	211 (36.9)	0.079	1.239 (0.976–1.574)

*n*: number of samples.

^a^ *P* < 0.05, significantly different, compared with the control group.

**Table 5:** Genotype and allele frequencies and odds ratios of MATN3 SNP6 (rs8176070) in control, cervical OA cases.

	Normal	All cervical OA	*P* ^ a^	OR (95% CI)
Genotype	*n* = 312 (%)	*n* = 228 (%)		
bb	149 (47.8)	108 (47.4)		
Bb	126 (40.4)	88 (38.6)	0.823	1.046 (0.708–1.545)
BB	37 (11.9)	32 (14.0)	0.137	1.534 (0.872–2.698)
Allele	*n* = 624 (%)	*n* = 456 (%)		
b	424 (67.9)	304 (66.7)		
B	200 (32.1)	152 (33.3)	0.657	1.060 (0.820–1.371)

*n*: number of samples.

^a^ *P* < 0.05, significantly different, compared with the control group.

**Table 6:** Genotype and allele frequencies and odds ratios of MATN3 SNP6 (rs8176070) in control, hand OA cases.

	Normal	All hand OA	*P* ^ a^	OR (95% CI)
Genotype	*n* = 312 (%)	*n* = 28 (%)		
bb	149 (47.8)	9 (32.1)		
Bb	126 (40.4)	17 (60.7)	0.002	5.380 (1.828–15.835)
BB	37 (11.9)	2 (7.1)	0.379	2.410 (0.340–17.084)
Allele	*n* = 624 (%)	*n* = 56 (%)		
b	424 (67.9)	35 (62.5)		
B	200 (32.1)	21 (37.5)	0.404	1.272 (0.722–2.241)

*n*: number of samples.

^a^ *P* < 0.05, significantly different, compared with the control group.
